# Clinical Implications of the Cervical Papanicolaou Test Results in the Management of Anal Warts in HIV-Infected Women

**DOI:** 10.1371/journal.pone.0081751

**Published:** 2013-11-28

**Authors:** Hung N. Luu, E. Susan Amirian, R. Palmer Beasley, Linda Piller, Wenyaw Chan, Michael E. Scheurer

**Affiliations:** 1 Dan L. Duncan Cancer Center, Baylor College of Medicine, Houston, Texas, United States of America; 2 Division of Epidemiology Human Genetics, and Environmental Sciences, School of Public Health, The University of Texas Health Science Center, Houston, Texas, United States of America; 3 Department of Pediatrics, Baylor College of Medicine, Houston, Texas, United States of America; 4 Division of Biostatistics, School of Public Health, The University of Texas Health Science Center, Houston, Texas, United States of America; University of Pennsylvania, United States of America

## Abstract

The Papanicolaou test (or Pap test) has long been used as a screening tool to detect cervical precancerous/cancerous lesions. However, studies on the use of this test to predict both the presence and change in size of genital warts are limited. We examined whether cervical Papanicolaou test results are associated with the size of the largest anal wart over time in HIV-infected women in an on-going cohort study in the US. A sample of 976 HIV-infected women included in a public dataset obtained from the Women’s Interagency HIV Study (WIHS) was selected for analysis. A linear mixed model was performed to determine the relationship between the size of anal warts and cervical Pap test results. About 32% of participants had abnormal cervical Pap test results at baseline. In the adjusted model, a woman with a result of Atypia Squamous Cell Undetermined Significance/Low-grade Squamous Intraepithelial Lesion (ASCUS/LSIL) had an anal wart, on average, 12.81 mm^2^ larger than a woman with normal cervical cytology. The growth rate of the largest anal wart after each visit in a woman with ASCUS/LSIL was 1.56 mm^2^ slower than that of a woman with normal cervical results. However, they were not significant (*P* = 0.54 and *P* = 0.82, respectively). This is the first study to examine the relationship between cervical Pap test results and anal wart development in HIV-infected women. Even though no association between the size of anal wart and cervical Pap test results was found, a screening program using anal cytology testing in HIV-infected women should be considered. Further studies in cost-effectiveness and efficacy of an anal cytology test screening program are warranted.

## Introduction

About 1% of sexually active adults in the United States have genital warts [1] and HIV-infected persons have higher prevalence and rate of recurrence of genital warts than HIV-uninfected persons [2,3]. Anal warts are a major public health problem for several reasons. They have never been studied separately from genital warts in prior research, and yet anal warts are found to be more common than cervical warts in women [4]. Additionally, a person infected with HPV is more likely to have multiple HPV types (i.e., high-risk and low-risk HPV), and there is evidence that the presence of anal warts may predict risk of anal intraepithelial neoplasia (AIN) or anal carcinoma [5,6], most likely as an indicator that unmeasured high-risk HPV types are present concurrently. Recently, a large Danish cohort study of approximately 50,000 patients found that there was a strong association between the presence of genital warts and anal cancer in men and women (standardized incidence ratio: 12.5 and 7.8 for men and women, respectively) [7]. And finally, a person with anal warts may experience serious financial and psychological burdens [8,9]. A recent study [10] showed that the annual cost to treat anogenital warts in the US is approximately $200 million (US). Patients with anogenital warts were reported to have decreased self-esteem and increased distress, embarrassment, shame, anger, negative self-perception and anxiety [9]. 

Previous research has indicated that the anal transformation zone (from anal squamous epithelium to rectal columnar epithelium) is morphologically similar to the cervical transformation zone, which is known to be susceptible to oncogenic HPV infection [11]. Also, anal cancer and cervical cancer have been found to share the same route of HPV infection [12]. Subsequent studies have shown that women with cervical dysplasia or cervical cancer are also at increased risk for anal cancer [13-15] and that concurrent HPV cervical infection has been identified as a risk factor for anal HPV infection [16,17]. Accordingly, a woman with cervical HPV infection is 20.5 times (95% CI: 16.3-25.7) more likely to have subsequent anal HPV infection with the same genotype compared to a woman without a previous cervical HPV infection [16]. 

Cervical Papanicolaou testing has long been used as a screening test to detect cervical precancerous/cancerous lesions. However, research on the use of this test to predict the presence of warts has been limited to genital warts only and no previous study has examined its association with anal warts. For example, in the early 1990s Rowen et al. [18] reported that women with warts (or sexual partners with genital warts) were more likely to have borderline or dyskaryotic smears than women without such a history. Recently, Kanno et al. [19] showed that women with genital warts were 7.03 times (95% confidence interval - CI: 2.82-17.53) more likely to have abnormal Papanicolaou test results than women without genital warts. From a clinical standpoint, it is interesting to ask whether the cervical Pap test might predict the development of anal wart size, given the widespread clinical use of this test. 

The purpose of the current study was to determine the association between abnormal cervical Papanicolaou test results and the size of the largest anal wart over time in HIV-infected women in an on-going cohort study in the US.

## Methods

### Study population

Data for this analysis were obtained from a public dataset (release P09) of the WIHS, an on-going cohort study of HIV-infected and HIV-uninfected women in 6 locations in the US: Bronx/Manhattan, NY; Brooklyn, NY; Washington DC; Los Angeles/Southern California/Hawaii; San Francisco/Bay Area, CA; and Chicago; IL. Details on the WIHS were reported previously [20,21]. Briefly, WIHS had 2 major enrollment periods: October 1994-November 1995, when 2,059 HIV-infected and 569 HIV-uninfected women were recruited from clinic-based and population-based sources; and October 2001-September 2002, when 1,144 women were recruited. All participants provided written informed consent to participate in the WIHS study, and the study was approved by the Institutional Review Boards of all participating institutions. Interviews, medical exams, gynecologic exams, specimen collections, and medical record abstraction were performed at the baseline visit and follow-up visits at 6-month intervals. 

**Figure 1:**
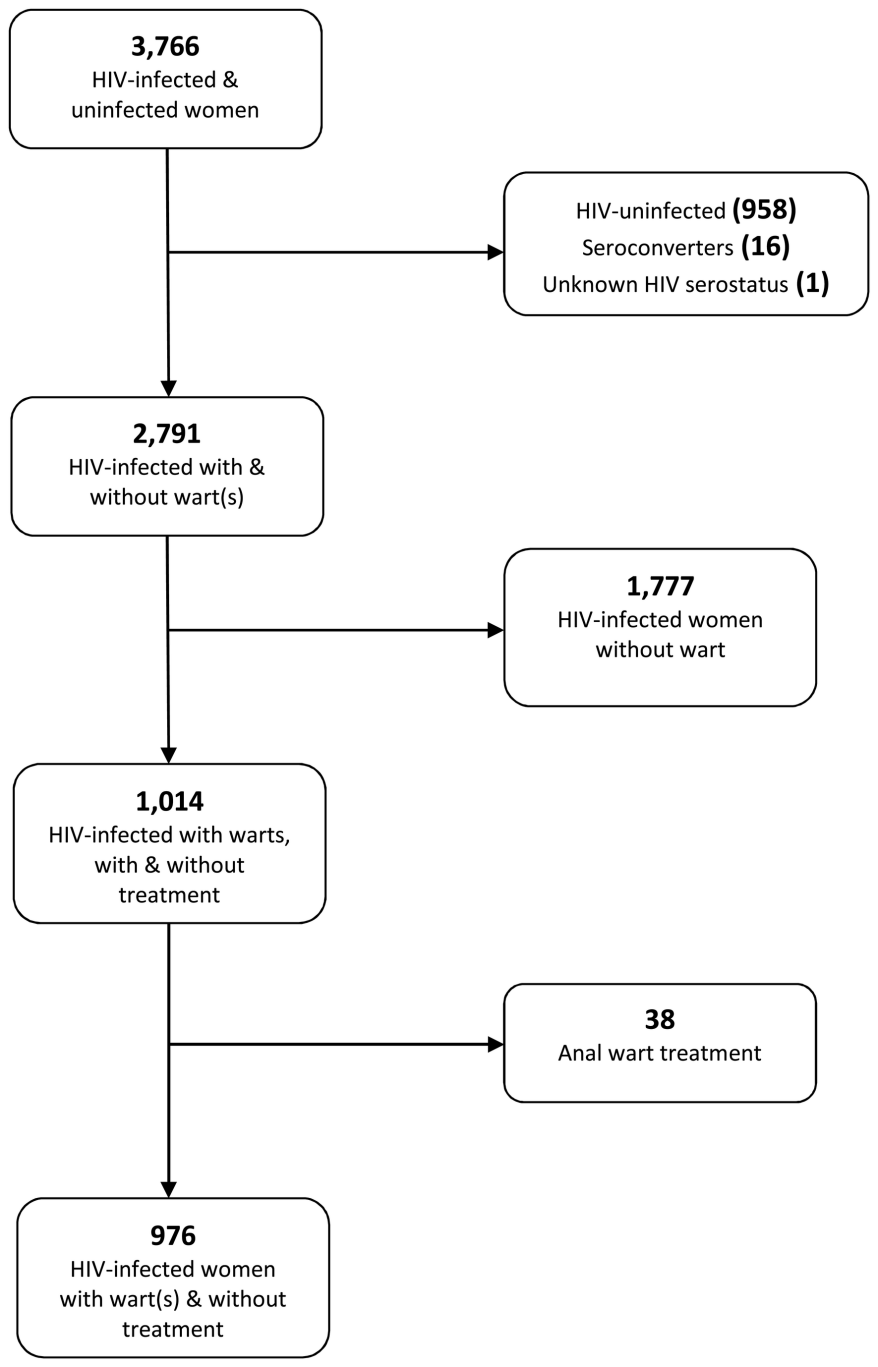
Flowchart of inclusion and exclusion of participants in current analysis.

The WIHS public dataset has 3,766 HIV-infected and HIV-uninfected women. A participant was included in the current analysis if she had at least one anal wart during follow-up. Participants were not included if they were HIV-uninfected (n = 958), seroconverted during the study follow-up (n = 16), had an unknown HIV status (n = 1), did not have at least one anal wart during the study period (n = 1,777), or received treatment for an anal wart during follow-up (n = 38). After these exclusions, a sub-sample of 976 participants was available for the current analysis (Figure 1). 

### Variables of interest and measurement

#### Outcome variable

In the current analysis, the outcome variable was the size of the largest anal wart present at a given visit. During the gynecologic examination, a trained examiner identified the presence of anal warts and then measured the largest anal wart in length and width (in millimeters). The presence of an anal wart was defined as warts in one of the following locations: “anus upper left”, “anus lower left”, “anus upper right”, “anus lower right”, “perineum left”, “perineum right”. The size of the anal wart was calculated by multiplying the length and the width of the reported largest anal wart. If multiple ano-genital warts were present, we assumed that the largest wart was an anal wart. 

#### Independent variables

The independent variable for the current analysis was cervical Papanicolaou test results. A wooden Ayres spatula and brush were used to obtain single-slide Papanicolaou samples. Cervical Papanicolaou test results were interpreted centrally at Danion (New York, NY; formerly Kyto or Kyto Meridien) using the 1991 Bethesda System [22,23]. Each Papanicolaou test was examined by two independent cytotechnologists. All specimens with abnormal cervical results (i.e., identified by either cytotechnologist) and 10% of specimens with negative results were evaluated by a third cytopathologist [24]. 

#### Other variables

The following potential confounders were collected at the baseline visit and follow-up visits. Race/ethnicity was categorized as African-American, Caucasian, and others for the purpose of the current analysis. The number of sex partners in the past six months was categorized as 0 and ≥1. Annual household income (≤$6,000, $6,000-$11,999, $12,000-$23,999, and ≥$24,000), marital status (married or living with partners, widowed, separated or divorced, and never married) and education level (less than high school education, high school education or GED, some college, and college graduate or graduate school) were also collected as part of the questionnaire. Highly active antiretroviral therapy (HAART) use (“Yes”/“No”) was defined at each visit if a woman met one of the following criteria: 1) Two or more nucleoside reverse transcriptase inhibitors (NRTIs) in combination with at least one protease inhibitor (PI) or one non-nucleoside reverse transcriptase inhibitors (NNRTI); 2) One NRTI in combination with at least one PI and at least one NNRTIs; 3) Regimen containing ritonavir and saquinavir in combination with one NRTI and no NNRTI; 4) An abacavir or tenofovir containing regimen of 3 or more NRTIs in the absence of both PI and NNRTIs, except for the three-NRTI regimens consisting of: abacavir + tenofovir + lamivudine or didanosine + tenofovir + lamivudine. A combination of zodovudine (AZT) and stavudine (d4T) with either a PI or NNRTI was not considered HAART. Monotherapy was defined as taking one NRTI, or only PI, or only NNRTI [25,26]. This definition of HAART is consistent with prior WIHS studies [27,28].

Two other important variables included in the current study were CD4+ cell count (<200, 200-500, >500 cells/mm^3^) and HIV viral load (<4,000, 4,000-20,000, 20,001-100,000, >100,000 copies/mL). These categorizations were used for consistency with previous WIHS studies [27,29,30]. CD4+ cell count was measured using flow cytometry at the laboratories certified by the AIDS Clinical Trial Groups [20]. Serum HIV viral load was measured using nucleic acid sequence-based amplification assay (NASBA) by Organon Teknika, Oklahoma City, OK at the laboratory of the National Institute of Allergy and Infectious Disease, AIDS Program, Virology Assurance HIV RNA Proficiency Program [20].

### Statistical analysis

We examined the distribution of socio-demographic characteristics. Means and standard deviations were calculated for continuous variables and counts and respective frequencies were calculated for categorical variables. For HIV viral load, a value of 10 copies/mL was used for those whose levels were undetectable [31].

A linear mixed model was used to identify the association between cervical Papanicolaou test results and the size of anal warts over time. In this model, the size of the largest anal wart was treated as the dependent variable and cervical Papanicolaou test results were treated as the independent variable. Cervical Papanicolaou test results were categorized as 1) normal, 2) atypical squamous cell undetermined significance (ASCUS)/low-grade squamous cell intraepithelial lesion (LSIL), and 3) high-grade squamous cell intraepithelial lesion (HSIL)/carcinoma. We decided to group HSIL/carcinoma because there are few cases in ASCUS (n=26), HSIL (n=22), and carcinoma (n=2) groups. The linear mixed model was chosen for the several reasons. It allowed us to deal with missing values, a common phenomenon in longitudinal studies. Furthermore, it deals effectively with a high correlation of repeated measurements within and between individuals. And finally, it resolves the difficulties of unbalanced measurements of subjects (i.e., number of visit in this study) and the time interval between measurements [32]. In the WIHS, the time interval between measurements was approximately 6 months; thus the unbalanced time interval was less of an issue in our analysis. 

A two-step model building process was employed. We first built an unadjusted model to determine the total variation of growth velocity and to check if each of the variables contributed significantly to the model [32]. We then built an adjusted model using the following covariates: number of sex partners in the past 6 months, marital status, enrollment period, education level, annual household income, and HAART use. In both unadjusted and adjusted models, we treated cervical Papanicolaou test results as a time-dependent variable. The following variables were also treated as time-dependent variables in the adjusted model: number of sex partners in the past 6 months, marital status, annual household income, HAART use, education level. These time-dependent variables entered the model both as a main effect and as a product with time (i.e., respective visit). Two time-independent variables were race/ethnicity and enrollment period (1 vs. 2) and entered the adjusted model as a main effect only. We did not include CD4+ cell count and HIV viral load into the final model because we found no association between them and the size of anal warts in previous analysis [33]. The PROC MIXED command of SAS 9.2 statistical package (Cary NC) was used in the modeling process [34]. All tests were two-sided and *P* = 0.05 was used as the significance level. 

## Results

At the baseline visit, more than 68% of participants in the current analysis had normal cervical Papanicolaou test results, 29% had either ASCUS or LSIL and only 3% had either HSIL or cervical carcinoma. While the proportion of Caucasian and the other race/ethnicity groups (i.e., Hispanic, Asian/Pacific Islanders, and Native America/Alaskan Native) were similar (19.42% and 19.94%, respectively), approximately 66% of HIV-infected women were African-American (Table 1). 

**Table 1:** Baseline Socio-demographic Characteristics of the WIHS HIV-infected Participants in the Current Study.

Characteristics	WIHS study (976) (n, %)
CD4+ cells count (cells/mm^3^)	
Mean CD4+ cell count±SD	324.59±293.04
<200	148 (19.8
200-500	328 (43.8)
>500	272 (36.4)
HIV RNA viral load (copies/mL)	
Mean viral load±SD	181,175±1,039,797
<4,000	331 (34.8)
4,000-20,000	164 (17.2)
>20,000-100,000	215 (22.6)
>100,000	242 (25.4)
Cytologic testing results	
Normal	530 (68.2)
ASCUS/LSIL	223 (28.7)
HSIL/Carcinoma	24 (3.1)
Age (Median±SD)	
≤25	66 (6.8)
26-35	383 (39.3)
36-45	407 (41.7)
>45	119 (12.2)
Ethnicity	
Caucasian American	189 (19.4)
African American	590 (60.6)
Others	194 (19.9)
Education	
<High school education	317 (36.4)
High school education or GED	295 (33.8)
Some college	207 (23.7)
College graduate or graduate school	53 (6.1)
Annual household income	
≤$6,000	125 (25.6)
$6,001-$12,000	171 (35.0)
$12,001-$24,000	118 (24.2)
≥24,001	74 (15.2)
Marital status	
Married or living with partner	245 (35.0)
Widowed	55 (7.9)
Separated or divorced	146 (20.9)
Never married	254 (36.3)
Number of male sex partners in the past 6 months	
0	259 (27.5)
≥1	682 (72.5)
HAART use at baseline	
No	285 (97.6)
Yes	7 (2.4)
Mean size of largest anal wart (mm^2^) ±SD^a^	13.65±127.71

Abbreviations: ASCUS, Atypical squamous cell undetermined significance; LSIL, Low grade squamous intraepithelial lesion; HSIL, high grade squamous intraepithelial lesion; GED, General education development; SD, Standard deviation.

^a^ Among those with anal warts at baseline (*n*=417)

Table 2 presents the results on the association between size of anal wart and cervical Papanicolaou test results over time, both in unadjusted and adjusted models. In the unadjusted model, the growth rate of the largest anal wart after each visit in a woman with cervical Papanicolaou test results of HSIL/carcinoma was 19.50 faster than that of a woman with a normal cervical Papanicolaou test result (*P* = 0.008). However, this finding was insignificant in the adjusted model (*P* = 0.56), possibly due to the small sample size in the HSIL/carcinoma group. In the adjusted model, a woman with Papanicolaou test results of ASCUS/LSIL had an anal wart, on average, 12.81 mm^2^ larger than a woman with normal cervical Papanicolaou test results. The growth rate of the largest anal wart after each visit in a woman with results of ASCUS/LSIL was 1.56 mm^2^ slower than that of a woman with a normal test result. These two results, however, were not statistically significant (*P* = 0.54 and *P* = 0.82, respectively). 

**Table 2:** Linear Mixed Model of Size of Anal Warts and Pap Smear Results in the WIHS HIV-infected Participants of the Current Study in Unadjusted and Adjusted Models.

	**Un-adjusted model**		Adjusted model^†^	
	Estimates ± SD	*P*-value	Estimates ± SD	*P*-value
Intercept	10.99±18.54	0.55	36.92±31.33	0.24
Visit	1.68±1.72	0.33	-1.76±6.79	0.80
Normal	Ref^a^		Ref^c^	
ASCUS/LSIL	38.65±29.23	0.19	12.81±12.39	0.31
HSIL/Carcinoma	-100.54±76.50	0.19	-12.86±44.80	0.78
Visit × (Normal)	Ref^b^		Ref^d^	
Visit × (ASCUS/LSIL)	0.96±2.63	0.72	-1.56±2.71	0.85
Visit × (HSIL/Carcinoma)	19.50±7.39	0.008	3.92±20.52	0.56

Abbreviations: ASCUS, Atypical squamous cell undetermined significance; HSIL, High grade squamous cell intraepithelial lesion; LSIL, Low grade squamous cell intraepithelial lesion; SE, Standard error.

^a^Type 3 *P*=0.13; ^b^Type 3 *P*=0.03; ^c^Type 3 *P*=0.54; ^d^Type 3 *P*=0.82;

^†^ Model adjusted for number of sex partners in the last 6 month, race/ethnicity, HAART use, enrollment, marital status, annual household income and education level.

## Discussion

In this study, we examined the association between cervical Papapicolaou smear results and the size of the largest anal wart over time in HIV-infected women using a public dataset from the largest on-going prospective cohort study in HIV-infected and HIV-uninfected women in the US. We found that women with abnormal cervical Papanicolaou test results (ASCUS/LSIL) had larger anal warts at baseline and slower growth rate of largest anal warts after each visit than those with normal results, although our results were not statistically significant. 

To our knowledge, this is the first study to use a linear mixed model to determine whether cervical Papanicolaou test results could predict the size of anal warts over time in HIV-infected women. We, therefore, cannot compare our findings directly to any other study. There are, however, a few studies reporting the relationship between the presence of genital warts and abnormal Papanicolaou tests in the general population [18,19]. For example, Kano et al.[19] reported that genital warts were significantly associated with abnormal Papanicolaou tests among HIV-uninfected women seen in a public STD clinic in Baltimore, MD (Odds Ratio - OR = 7.03; 95% CI: 2.82-17.53; *P*<0.001). We noted that there are two major differences between our findings and those reported by Kano et al. [19]. First, the outcome variable in our study was the size of the largest anal wart, while Kano et al. [19] examined abnormal Pap smear results. Second, in our study, the size of anal warts was examined over time, whereas Kano et al. [19] examined the presence of a genital wart regardless of the size.

Although we did not find a significant association between cervical Papanicolaou test results and the size of anal warts over time among HIV-infected women, this study provides some clinical implications for future screening programs of HIV-infected women. The cervical Papanicolaou test has contributed to the reduction of cervical cancer prevalence and incidence in developed countries. With regard to screening for anal HPV infections, pre-cancerous/cancerous lesions and warts, a reliable test with high accuracy is still needed. In a small subsample from WIHS, Holly et al. [35] did not find concurrent cervical cytology to be a useful overall indicator of anal cytology (Relative risk-RR = 1.5; 95% CI: 0.97-2.2). Also, using anal Papanicolaou tests to evaluate a subset of the WIHS cohort (223 HIV-infected and 57-uninfected women), Palefsky et al. [36] reported that 76% and 42%, respectively, had anal HPV infection, and 26% and 8%, respectively, had abnormal anal Papanicolaou test results. These findings, together with our own, demonstrate that the cervical Papanicolaou test might not be predictive of the behavior of anal warts, which are very common among HIV-infected women. Instead, anal Papanicolaou testing may be a relevant approach in this population. 

Previous studies have indicated that HIV-infected women are more likely to have abnormal anal cytology/histology than HIV-uninfected women [37,38]. Recent local guidelines in New York recommended anal Papanicolaou screening be conducted in men who have sex with men (MSM), females with abnormal vaginal and/or vulvar histology, and any patients with a history of anogential condylomas [39]. This guideline, however, did not recommend anal Papanicolaou screening in HIV-infected women with no other pathological abnormalities. Our findings, along with the prior evidence, support a recommendation for the use of anal Papanicolaou test screening for HIV-infected women, given the fact that anal Papanicolaou testing has proven to be cost-effective in the HIV-infected MSM population [40] and has fairly comparable accuracy to cervical cytology [41]. 

The current analysis has two major strengths. First, the linear mixed model allowed us to address the research question directly regarding whether or not there is an association between the size of anal warts and cervical Papanicolaou test results over time while other statistical methods cannot. Second, the size of the largest anal wart, as the continuous outcome variable, was treated appropriately. If we had used other statistical methods such as logistic regression or Cox proportional hazards regression, we could not model the change in size of anal warts over time. A limitation in our study is the use of the size of the largest anal wart as an outcome variable. Since the largest wart measured at one visit might not be the same in subsequent visits, it is difficult to follow-up the same anal wart over time. We, however, thought that the overall disease burden posed by anal warts may well depend on the largest one present at any given visit.

In summary, we found that women with abnormal cervical Papanicolaou test results (ASCUS/LSIL) had larger anal warts at baseline and slower growth rate of the largest anal warts than those with normal results. The association between the size of anal warts and cervical Papanicolaou test results over time in HIV-infected women, however, becomes insignificant in the adjusted model. Our findings, however, provide more information for further consideration of screening programs using anal Papanicolaou testing in HIV-infected women. Currently, data on the natural history of anal HPV infection and its clinical endpoints (both warts and precancerous lesions) in HIV-infected women are limited. Additional studies among this particular population are required to address this question and support the implementation of an anal Papanicolaou screening program.
